# GPR174 signals via G*α*s to control a CD86-containing gene expression program in B cells

**DOI:** 10.1073/pnas.2201794119

**Published:** 2022-05-31

**Authors:** Elise W. Wolf, Zachary P. Howard, Lihui Duan, Hanson Tam, Ying Xu, Jason G. Cyster

**Affiliations:** ^a^HHMI, University of California, San Francisco, CA 94143;; ^b^Department of Microbiology and Immunology, University of California, San Francisco, CA 94143

**Keywords:** G-protein–coupled receptor, B lymphocytes, GPR174, CD86, NUR77

## Abstract

GPR174 is abundantly expressed in B and T lymphocytes and has a role in restraining T cell responses, but the function of GPR174 in B cells is less clear. Here we report that upon in vitro culture B cells undergo a spontaneous GPR174-dependent activation process that is associated with marked changes in gene expression, including up-regulation of *Cd86*, *Nr4a1*, *Ccr7*, and phosphodiesterases. B cells lacking G*α*s show a block in induction of the GPR174-dependent program. Spontaneous up-regulation of CD86 in cultured B cells is dependent on protein kinase A. Both GPR174- and G*α*s-deficient B cells show enhanced survival in culture. In vivo, GPR174 contributes to NUR77 expression in follicular B cells and is needed for establishing a marginal zone compartment of normal size. Treatment of mice with lysophosphatidylserine (lysoPS), a GPR174 ligand, is sufficient to promote CD86 up-regulation by follicular B cells. These findings demonstrate that GPR174 can signal via G*α*s to modulate B cell gene expression and show this can occur in vivo in response to lysoPS. Additionally, the findings illuminate a pathway that might be targeted to improve systems for the in vitro study of B cell responses.

## Significance

Modeling immune responses in vitro is critical for studying many facets of the B cell response. We show that during culture without stimulation, mouse B cells undergo massive changes in gene expression. Many of these changes are promoted by GPR174 signaling via G*α*s. GPR174 and G*α*s also contribute to reduced B cell viability during culture. We suggest that GPR174 antagonists may be useful to reduce the shift in gene expression and to augment B cell survival during culture. We also provide evidence that ligand engagement of GPR174 can activate this pathway in vivo. Variants in the GPR174 locus have been associated with autoimmune diseases. Our findings provide knowledge for understanding how alterations in GPR174 expression may contribute to disease.

G-protein–coupled receptors (GPCRs) comprise a large family of seven-transmembrane signaling proteins, many of which have roles in the development and function of the mammalian immune system. The outcome of GPCR signaling is determined via coupling to various G proteins. Signaling via G*α*i-coupled GPCRs promotes cell migration, whereas GPCR coupling to G*α*12/13 proteins often leads to migration inhibition. In contrast, G*α*s signaling exerts its effect through cAMP production and leads to complex and often suppressive effects on immune cell activation and proliferation. GPCR GPR174 is one of several related X-linked receptors that are highly expressed on lymphocytes (1–3). Several genome-wide association studies (GWAS) have linked *GPR174*-associated single-nucleotide polymorphisms (SNPs) to autoimmune disorders including Graves’ and Addison’s diseases (4–6).

GPR174 was found to be a receptor for lysophosphatidylserine (lysoPS) and was suggested to signal via G*α*12/13-containing heterotrimeric G proteins (1). Studies on GPR174-deficient mice revealed a role for the receptor in restraining T regulatory (Treg) cell development and function (2) and conventional T cell proliferation and IL-2 production (7, 8). In these reports, signaling was suggested to occur via G*α*s-containing heterotrimeric G proteins (7, 8). In another study, GPR174 was reported to support B cell migration to spleen stromal cell culture supernatants and biochemical fractionation led to the suggestion that CCL19 and CCL21, well-defined CCR7 ligands, were ligands for GPR174 (3). In that study, GPR174 was suggested to signal via G*α*i and G*α*13 (3). Taken together, there is currently a lack of clear understanding regarding the signaling pathway(s) engaged downstream of GPR174.

CD86 is a critical costimulatory molecule in antigen-presenting cells including B cells (9, 10). Induction of CD86 by B cell receptor (BCR) signaling is an important feature of the BCR-induced activation program, although CD86 can also be induced in B cells by CD40 and cytokine signaling (11–13). Interestingly, *Cd86* was originally characterized as a cAMP-inducible gene in B cells (14–18). However, since the BCR, CD40, and cytokines are not thought to induce CD86 via cAMP, the receptors leading to cAMP-mediated induction of CD86 in B cells have been unclear.

The study of B cells in vitro has been critical to numerous advances in the understanding of adaptive immunity and it remains a crucial method for dissecting the B cell response. Although not widely reported on, it is generally understood by B cell biologists that B cells undergo some amount of gene expression change during in vitro culture. Indeed, this aspect of cultured B cells presented a major challenge to efforts by the Alliance for Cell Signaling (19, 20) to dissect the signaling circuits in mouse B cells. The pathways involved in causing these gene expression changes are not understood.

Here we report that cultured follicular B cells rapidly up-regulate CD86 independently of stimulation in a GPR174- and G*α*s-dependent manner. The GPR174-mediated response was not dependent on exogenous ligand or BCR signaling. RNA-sequencing analysis revealed that B cells underwent changes in expression of 1,000 genes after 4 h of unstimulated culture and many of these changes were GPR174 dependent. This included induction of *Cd86*, *Nr4a1*, *Ccr7*, and phosphodiesterases and down-regulation of immunoreceptor tyrosine-based inhibitory motif (ITIM)-containing receptors. There was a strong overlap in the alterations in gene expression between GPR174- and G*α*s-deficient B cells, indicating that GPR174 signals via G*α*s in follicular B cells. When B cells were maintained in vitro for 1 to 2 d in the absence of stimulation, GPR174 and G*α*s deficiencies were associated with augmented survival. In vivo GPR174 deficiency led to reduced NUR77-GFP reporter expression, and GPR174- and G*α*s-deficient mice had a reduced marginal zone (MZ) B cell compartment. Treatment of mice with lysoPS was sufficient to cause increased CD86 expression by follicular B cells. These findings establish GPR174 as a receptor capable of exerting a large influence on B cell gene expression and they provide insight into the B cell properties that can be influenced by the receptor.

## Results

### GPR174-Deficient B Cells Have a Defect in CD86 Up-Regulation in Vitro.

**Fig. 1.**
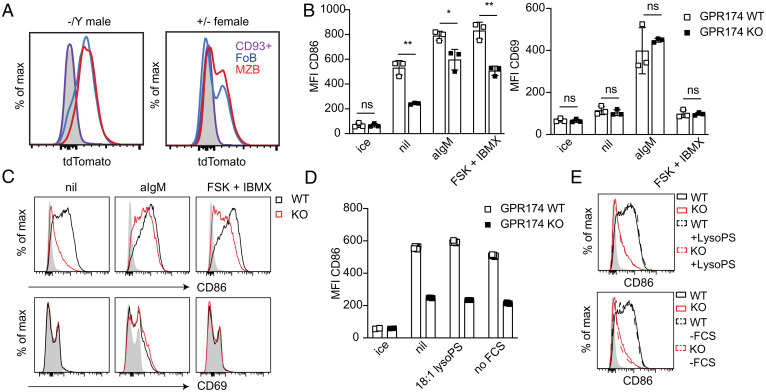
GPR174 promotes CD86 induction. (*A*) Representative tdTomato reporter expression in *Gpr174^--∕Y ^*and *Gpr174^+∕--^* mice for transitional B (CD23^--^CD93^+^), splenic follicular B (FoB), and marginal zone B cells (MZB) versus reporter negative (gray). (*B*) Summary MFI data of CD86 (*Left*) and CD69 (*Right*) on B cells cultured for 6 h with or without the indicated stimuli or maintained on ice (*n* = 3 mice per genotype). (*C*) Representative histogram plots of the data presented in *B* with the gray histograms corresponding to WT cells kept on ice. (*D*) Mean fluorescence intensity (MFI) of CD86 on B cells of the indicated genotypes (triplicate wells) incubated for 6 h in normal media (nil), media with 18:1 lysoPS (10 µM), media without FCS, or maintained on ice. (*E*) Representative histograms of the data presented in *D* versus WT cells maintained on ice (gray). Bar graphs represent mean ± SD. *B* and *D* are representative of at least three experiments including both male and female mice. Statistical significance for *B* was determined by unpaired *t* test. ns, not significant; *P < 0.05; **P < 0.01.

To examine the role of GPR174 in B cell activation, we utilized mice in which the single coding exon of *Gpr174* was replaced with an in-frame tdTomato allele. These mice show high reporter expression in naive T and B cells (2). In accordance with Immunological Genome Project (Immgen) RNA sequencing (RNAseq) data of *Gpr174* expression, there was high reporter expression in spleen follicular and MZ B cells, while minimal expression was observed in CD23^--^CD93^+^ transitional B cells (Fig. 1*A*). B cell reporter expression in *Gpr174^+∕--^* female mice, in which random X inactivation is predicted to yield 50% reporter positive (and thus GPR174-deficient) cells, displayed a bimodal population in follicular and MZ B cells, with minimal expression in early transitional B cells (Fig. 1*A*).

To determine whether GPR174 plays a role in the initial response to activating signals we performed in vitro stimulations of wild-type (WT) and GPR174-deficient B cells and analyzed the expression of surface markers by flow cytometry after 6 h of culture. As anticipated, the activation markers CD86, CD69, and CD83 were strongly induced within 6 h by BCR ligation with anti-IgM compared to cells kept on ice (Fig. 1*B* and *SI Appendix*, Fig. S1*A*). However, in contrast to CD69 and CD83, which were up-regulated to a similar extent in WT and GPR174-deficient B cells, CD86 (B7-2) up-regulation was significantly reduced in GPR174-deficient cells (Fig. 1*B*). Stimulation with anti-CD180 revealed a comparable defect in CD86 up-regulation in GPR174-deficient B cells (*SI Appendix*, Fig. S1*B*). In the course of these studies, we noted that substantial CD86 up-regulation occurred in B cells cultured in the absence of added stimulation and that this up-regulation was strongly GPR174 dependent (Fig. 1 *B* and *C*). In contrast, CD69 was not spontaneously up-regulated (Fig. 1 *B* and *C*) and CD83 was minimally induced (*SI Appendix*, Fig. S1*A*). Similar findings were made using cultures of total splenocytes or lymph node cells (*SI Appendix*, Fig. S1*C*) and with sorted B cells (*SI Appendix*, Fig. S1*D*) indicating that the CD86 induction was not dependent on factors from non-B cells. The effects of GPR174 expression were cell intrinsic, as gating on reporter-positive and -negative B cells from *Gpr174^+∕--^* female mice showed less CD86 induction in the reporter^+^ cells (that lack GPR174), with similar levels of CD69 (*SI Appendix*, Fig. S1 *E* and *F*). TdTomato reporter expression was comparable between cultured cells and cells kept on ice and was unaffected by the various stimulations (*SI Appendix*, Fig. S1*G*).

Engagement of G*α*s-coupled GPCRs drives cAMP generation, and CD86 is cAMP inducible (14–18). Forskolin induces cAMP production by adenylyl cyclase and 3-isobutyl-1-methylxanthine (IBMX) prevents cAMP degradation by phosphodiesterases (21, 22). As anticipated, combined treatment of B cells with forskolin and IBMX led to induction of CD86, but not of CD69 or CD83 (Fig. 1 *B* and *C* and *SI Appendix*, Fig. S1*A*). This treatment did not fully rescue the defect in CD86 induction in GPR174-deficient B cells. We suspect that this reflects a reduced ability of forskolin to engage adenylyl cyclase in cells that lack a dominant G*α*s-coupled receptor, as we discuss further below.

LysoPS is a ligand for GPR174 (1, 2, 7, 8, 23, 24). Addition of lysoPS to the B cell cultures did not alter CD86 induction (Fig. 1 *D* and *E*). To test whether this might be due to the presence of lysoPS in serum, we cultured B cells in the absence of fetal calf serum (FCS). Cells cultured under these conditions spontaneously induced CD86 to a comparable level to that of cells cultured in FCS-containing media (Fig. 1 *D* and *E*).

We also examined the effect of GPR174 deficiency in a lipopolysaccharide (LPS)-induced B cell proliferation assay. WT and GPR174-deficient B cells proliferated equivalently after 4 d and addition of lysoPS to the culture medium had no effect on the response (*SI Appendix*, Fig. S1*H*). This contrasted with CD4 T cells stimulated with anti-CD3 and anti-CD28, where lysoPS repressed CD4 T cell proliferation in a GPR174-dependent manner (*SI Appendix*, Fig. S1*H*) as previously observed (2). These discrepant effects on B and T cells may in part be explained by differences in GPR174 expression. LPS-stimulated B cells down-regulated the tdTomato reporter after 4 d (*SI Appendix*, Fig. S1*I*) in accord with plasma cells and germinal center (GC) B cells having minimal expression of the *Gpr174* locus (https://www.Immgen.org), whereas CD4 T cells activated with anti-CD3 and anti-CD28 maintained tdTomato reporter expression at similar levels to unstimulated cells (*SI Appendix*, Fig. S1*I*).

We considered the possibility that exogenous lysoPS failed to augment CD86 in unstimulated B cells because endogenous lysoPS increased in B cells during culture. Indeed, when lysoPS was quantitated in cultured B cells using liquid chromatography and mass spectrometry (LC-MS/MS), the amounts of 18:0 lysoPS were increased within 10 min of incubation and remained elevated for at least 60 min (*SI Appendix*, Fig. S1*J*).

### GPR174 Induction of CD86 Is Dependent on G*α*s and PKA and Is BCR Independent.

**Fig. 2.**
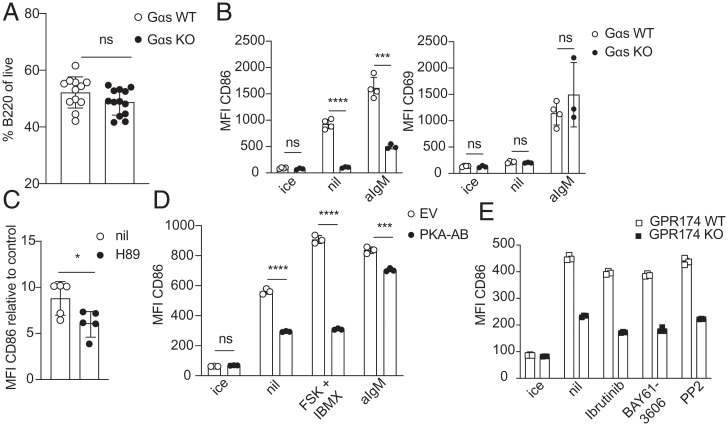
GPR174-dependent CD86 induction is G*α*s and PKA dependent and BCR independent. (*A*) Percentage of B220^+^ cells in the spleens of *Gnas^+∕+^ CD19 Cre^+∕--^* (G*α*s WT, *n* = 12) and *Gnas^fl∕fl^ CD19 Cre^+∕--^* (G*α*s KO, *n* = 13) mice. (*B*) MFI of CD86 (*Left*) and CD69 (*Right*) on G*α*s WT (*n* = 4 mice) or G*α*s KO (*n* = 3 mice) splenic follicular B cells cultured with or without anti-IgM. (*C*) MFI of CD86 on WT B cells cultured with or without 10 µM H89 relative to the MFI for cells maintained on ice. (*D*) MFI of CD86 on vector^+^ (gating shown in *SI Appendix*, Fig. S2*A*) B cells from chimeras transduced with EV or PKA-AB constructs and cultured with or without anti-IgM or forskolin + IBMX or maintained on ice (triplicate wells, from one experiment representative of three). (*E*) MFI of CD86 on B cells of the indicated genotype treated with or without Ibrutinib (1 µM), BAY61-3606 (1 µM), or PP2 (20 µM) (triplicate wells). All cultures were for 6 h. Bar graphs represent mean ± SD. *A*, *B*, and *E* are representative of at least three experiments including both male and female mice. Statistical significance for *B*–*D* was determined by unpaired *t* test. ns, not significant; *P < 0.05; ***P < 0.001; ****P < 0.0001.

Given prior studies indicating that CD86 is a cAMP-inducible gene (14–18) and work in T cells suggesting GPR174 function was G*α*s dependent (7), we asked whether *Gnas*, the gene encoding G*α*s, was required for GPR174 signaling in B cells. For these studies, we crossed *Gnas* floxed mice (25) with *Cd19-Cre* mice (26), yielding mice that conditionally lacked *Gnas* in B cells beginning in early development. G*α*s-deficient (*Gnas^fl∕fl^ Cd19-Cre^+∕--^*) mice had a comparable percentage of B220^+^ cells in the spleen to G*α*s WT (*Gnas^+∕+^ Cd19-Cre^+∕--^*) mice (Fig. 2*A*). As above, WT B cells up-regulated CD86 spontaneously over 6 h of culture. Importantly, G*α*s-deficient B cells did not up-regulate CD86 above cells kept on ice, representing an even stronger phenotype than observed in GPR174-deficient mice (Fig. 2*B*). G*α*s-deficient B cells also displayed a defect in CD86 induction in response to anti-IgM treatment while CD69 induction occurred normally (Fig. 2*B*). Forskolin binding to and activation of adenylyl cyclase and subsequent cAMP generation are dependent on G*α*s proteins (21). Accordingly, forskolin and IBMX treatment of G*α*s-deficient B cells did not induce CD86 (*SI Appendix*, Fig. S2*A*).

G*α*s activation leads to elevations of cAMP and subsequent activation of the cAMP-dependent kinase, PKA (27). To test for involvement of PKA in spontaneous CD86 up-regulation we cultured B cells in the presence of H89, a PKA inhibitor. H89 treatment partially blocked spontaneous CD86 up-regulation (Fig. 2*C*). Pharmacological inhibitors of PKA are known to have off-target effects (28). The regulatory type I subunit of PKA has two cAMP-binding sites, A and B, and mutation of these sites yields a dominant negative PKA (29). To confirm that the effect of H89 on spontaneous CD86 induction was via PKA, we generated retroviral bone marrow (BM) chimeras expressing the dominant negative PKA-AB construct or empty vector (EV), and a GFP reporter, in hematopoietic cells. Following 8 wk of reconstitution, we cultured splenocytes from these chimeras for 6 h either in media alone or with forskolin and IBMX. Compared to EV GFP^+^ B cells, PKA-AB GFP^+^ B cells induced less CD86 (Fig. 2*D*), suggesting that CD86 up-regulation during unstimulated culture is PKA dependent. This defect was most dramatic in the highest GFP-expressing PKA-AB cells, which showed a near-total loss of CD86 induction (*SI Appendix*, Fig. S2*B*). Expression of PKA-AB GFP also blocked all CD86 induction in response to forskolin and IBMX treatment and partially reduced CD86 induction following anti-IgM treatment (Fig. 2*D* and *SI Appendix*, Fig. S2*B*). These data suggest that GPR174 and G*α*s signal at least in part via PKA to induce CD86.

Since BCR signaling is a strong inducer of CD86, we wanted to determine whether a BCR-derived signal was required for spontaneous CD86 induction. Inhibition of Btk-, Syk-, or Src-family kinases, critical kinases downstream of the BCR (30), had a minimal effect on spontaneous CD86 induction (Fig. 2*E*). These inhibitors strongly inhibited anti-IgM–induced CD86 up-regulation, confirming their efficacy (*SI Appendix*, Fig. S2*C*).

We tested whether a similar pathway operated in human B cells by culturing peripheral blood mononuclear cells (PBMCs) for 6 h. Human B cells showed increased CD86 expression in the absence of stimulation and this induction was significantly inhibited by PKA antagonism (*SI Appendix*, Fig. S2*D*).

### RNAseq of Cultured WT and GPR174-Deficient B Cells Reveals Numerous Expression Changes.

**Fig. 3.**
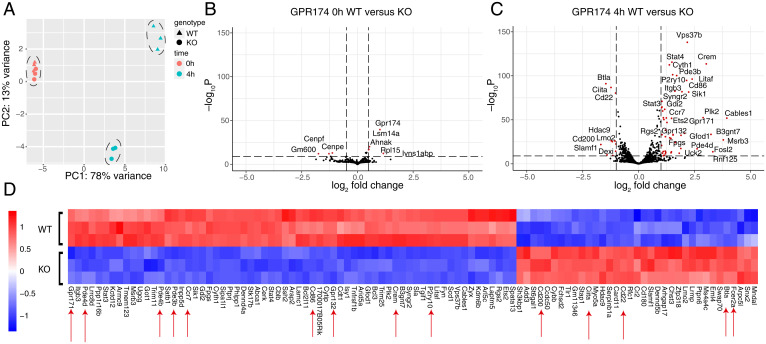
RNAseq of cultured WT and GPR174-deficient B cells reveals numerous expression changes. (*A*) PCA plot of RNAseq data from sorted GPR174 WT and KO B cells cultured for the indicated times (*n* = 3 mice per genotype). (*B* and *C*) Volcano plots of DEGs between WT and KO B cells at 0 h (*B*) and 4 h (*C*). Dashed lines indicate fold-change ($\left|\log_{2}\mathrm{FC}\right|>0.5$ for *B*, $\left|\log_{2}\mathrm{FC}\right|>1$ for *C*) and *P*-value cutoffs (*P*_adj _< 1 × 10^--10^ for highlighted DEGs in red. (*D*) Heatmap of top 100 DEGs in GPR174 WT versus KO follicular B cells after 4 h of culture. Red arrows indicate genes referenced in the text.

To investigate the full extent of the gene expression program regulated by GPR174 in cultured B cells we performed RNA sequencing on mouse follicular B cells immediately postsort (0 h) or after 4 h of culture without stimulation (31). Immediately ex vivo there were very few differences between WT and GPR174-deficient follicular B cells (Fig. 3 *A* and *B*), whereas gene expression diverged considerably after 4 h of culture as observed by principal-component analysis (Fig. 3 *A* and *C*). Importantly, cultured WT B cells underwent a massive change in gene expression compared to uncultured WT cells, with over 1,000 genes up- or down-regulated (*SI Appendix*, Fig. S3*A* and Dataset S1). Cultured GPR174-deficient B cells underwent many fewer changes compared to their uncultured counterparts (*SI Appendix*, Fig. S3*B* and Dataset S1). The top 100 differentially expressed genes (DEGs) that either increased or decreased expression from 0 to 4 h in WT B cells were highly enriched for genes that were increased in expression in WT compared to GPR174-deficient B cells at 4 h (*SI Appendix*, Fig. S3*C*). The top 100 DEGs that either increased or decreased from 0 to 4 h in GPR174-deficient B cells were less enriched for differential gene expression between WT and knockout (KO) B cells at 4 h (*SI Appendix*, Fig. S3*D*). Together, these data indicated that the majority of differential gene expression came from increased or decreased expression in WT cells, whereas GPR174-deficient cells were comparatively static.

After 4 h of culture, WT B cells more strongly expressed genes indicative of cAMP signaling than GPR174-deficient B cells, including the CREB family member cAMP response element modulator (*Crem*) and the cAMP-specific phosphodiesterases *Pde3b*, *Pde4b*, and *Pde4d* (Fig. 3*D*). As expected, *Cd86* was also highly differential (Fig. 3*D*). WT B cells showed elevated expression of the chemokine receptor *Ccr7* and the additional G-protein–coupled receptors *Gpr171*, *Gpr132*, and *P2ry10* (Fig. 3*D*). Other DEGs that showed higher expression in WT than GPR174-deficient B cells at the 4-h timepoint included immediate early response gene 2 (*Ier2*) and the *Nr4a* family of orphan transcription factors *Nr4a1* (encoding NUR77), *Nr4a2*, and *Nr4a3*, as well as the AP-1 transcription factors *Junb* and *Fosl2* (Dataset S1). In contrast, GPR174-deficient B cells had higher expression of inhibitory signaling molecules like *Btla*, *Cd22*, and *Cd200*, as well as class II MHC transactivator, *Ciita*, and *Fcer2a* (encoding CD23) than WT B cells after 4 h of culture (Fig. 3 *C* and *D*). Notably these genes were reduced in expression in WT cells and unchanged or less reduced in expression in GPR174-deficient cells compared to uncultured cells (Dataset S1).

We wondered whether expression of early response genes, such as *Ier2* and *Nr4a1*, suggested multiple waves of gene expression, with some expression changes less proximal to a GPR174-derived signal. To determine this, we performed RNA sequencing on sorted follicular B cells that had been cultured for 1 h (31). By 1 h of culture, many genes were already differential, although fewer than at the 4-h timepoint (Datasets S1 and S2). WT B cells displayed higher expression of *Pde4b*, *Nr4a* family members, AP-1 family members, *Crem*, *Cd86*, and *Ccr7*, whereas few genes had significantly higher expression in GPR174-deficient B cells (*SI Appendix*, Fig. S3 *E* and *F*). These data suggest that very rapid signaling occurs via GPR174 in cultured B cells to drive early gene expression changes.

### G*α*s-Deficient B Cells Phenocopy Gene Expression Changes in GPR174-Deficient Cells.

**Fig. 4.**
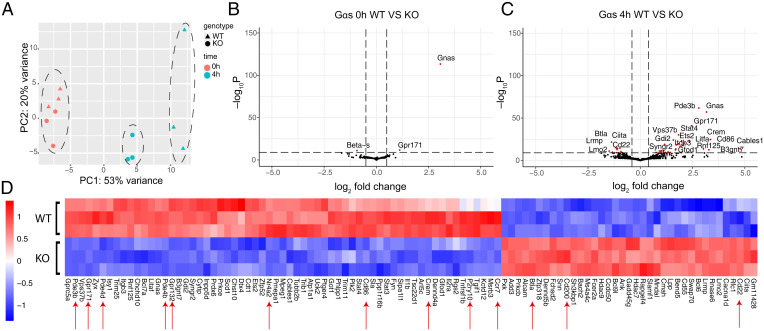
G*α*s-deficient B cells phenocopy gene expression changes in GPR174-deficient cells. (*A*) PCA plot of RNAseq data from sorted G*α*s WT and KO B cells cultured for the indicated times (*n* = 3 mice per genotype). (*B* and *C*) Volcano plots of DEGs between WT and KO B cells at 0 h (*B*) and 4 h (*C*). Dashed lines indicate fold-change and *P*-value cutoffs for highlighted DEGs. (*D*) Heatmap of top 100 DEGs in G*α*s WT versus KO B cells after 4 h of culture. Red arrows indicate genes referenced in the text.

To further explore whether GPR174 signaling in B cells was G*α*s dependent, we performed RNA sequencing on sorted follicular B cells from G*α*s WT and G*α*s-deficient mice after 0 or 4 h of culture without stimulation (31). As for GPR174 deficiency, very few genes were differentially expressed between WT and G*α*s-deficient B cells immediately ex vivo (Fig. 4 *A* and *B*), whereas large changes occurred after 4 h (Fig. 4 *A* and *C*). Following 4 h of culture, WT and G*α*s-deficient B cells showed a strikingly similar pattern of divergent gene expression to WT and GPR174-deficient B cells. G*α*s WT B cells had increased expression of *Cd86*, *Crem*, phosphodiesterases, *Nr4a* family members, *Ccr7*, *Gpr132*, and *Gpr171* compared to G*α*s-deficient B cells (Fig. 4*D*). *P2ry10*, which is adjacent to *Gpr174* on the X chromosome, was increased in WT compared to G*α*s-deficient B cells, indicating that the *P2ry10* increase observed in WT versus GPR174-deficient B cells was not due to disruption of the locus in generating the *Gpr174 tdTomato* allele. Similar to GPR174-deficient B cells, G*α*s-deficient B cells had higher expression of *Cd22*, *Btla*, and *Cd200* than WT cells (Fig. 4*D*). As expected, G*α*s WT B cells showed similar gene induction to GPR174 WT B cells during culture (*SI Appendix*, Fig. S4*A*). Similar to GPR174-deficient B cells, G*α*s-deficient B cells displayed fewer changes in gene expression, and the changes that did occur overlapped with those in WT cells (*SI Appendix*, Fig. S4*B*).

Interestingly, expression of *Ier2* and *Gpr171*, which had increased expression in WT versus GPR174-deficient B cells at 4 h and both of which are predicted to contain conserved TATA-associated CRE sites (32), was higher in G*α*s WT than in G*α*s-deficient B cells immediately ex vivo (Dataset S3).

Gene set enrichment analysis (GSEA) of DEGs in WT versus G*α*s-deficient B cells at 4 h revealed a strong enrichment for differential expression in the WT versus GPR174-deficient 4-h dataset (*SI Appendix*, Fig. S4*C*). Furthermore, analysis of de novo transcription factor-binding motifs in DEGs that increased in GPR174 or G*α*s WT B cells during culture showed significant enrichment for CREB-binding motifs, whereas this was not the case for de novo motif analyses of GPR174- or G*α*s-deficient B cells (*SI Appendix*, Fig. S4*D*). The level of similarity between GPR174- and G*α*s-dependent transcript induction indicated that GPR174 was the primary G*α*s-coupled receptor in this context, despite the presence of additional G*α*s-coupled GPCRs in B cells, including ADRB2 and PTGER4 (33, 34) (https://www.Immgen.org). *Pde3b* was one gene that was more strongly affected by G*α*s than GPR174 deficiency, as it was moderately up-regulated in GPR174-deficient but not in G*α*s-deficient B cells (*SI Appendix*, Figs. S3*B* and S4*B*). PDE3b is activated by PKA phosphorylation (35), and G*α*s-mediated induction of this gene may be part of a negative feedback loop when cAMP is present.

Among the genes that increased in expression over time in GPR174- and G*α*s-deficient B cells, most increased to a lesser extent than in WT cells. However, there was a small set of genes that increased in expression more in GPR174- and G*α*s-deficient than in WT cells during culture. Comparing overlap of these DEGs revealed multiple genes involved in cholesterol uptake and biosynthesis, including *Ldlr*, *Sqle*, and *Hmgcs1* (*SI Appendix*, Fig. S4*E*). This may reflect a G*α*s-dependent repression of a latent cholesterol synthesis drive and could have relevance for understanding the repressive effect of some G*α*s-coupled receptors on lymphocyte proliferation (2, 7, 13, 36).

### Increased Survival of GPR174- and G*α*s-Deficient B Cells in Culture.

**Fig. 5.**
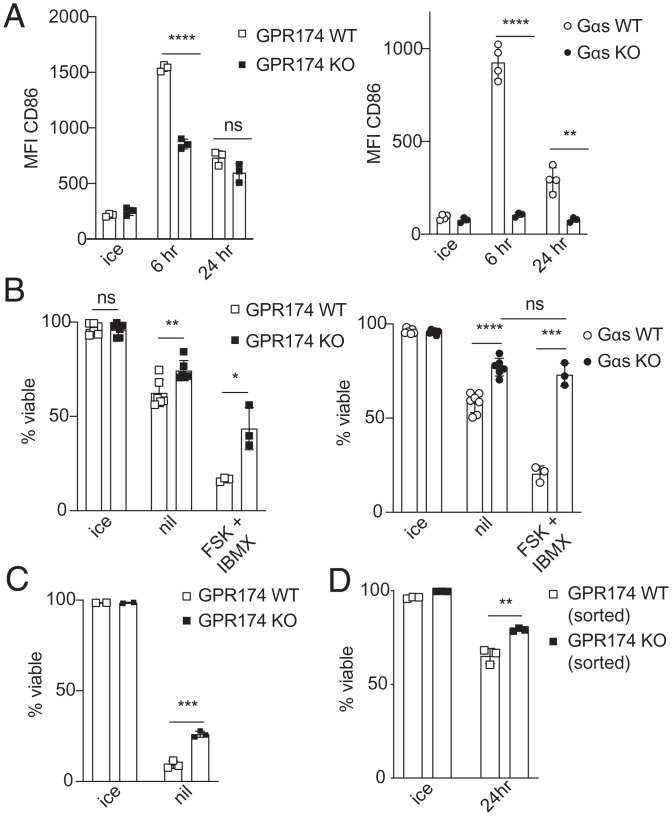
GPR174- and G*α*s-deficient B cells show increased viability in vitro. (*A*) MFI of CD86 on GPR174 (*Left*, *n* = 3 mice per genotype) or G*α*s (*Right*, *n* = 4 WT, *n* = 3 KO mice) B cells after 6 or 24 h of unstimulated culture. (*B*) Percentage of viable GPR174 (*Left*, *n* = 6 mice per genotype for ice/nil, *n* = 3 mice per genotype for FSK + IBMX) or G*α*s (*Right*, *n* = 7 WT, *n* = 6 KO for ice/nil, *n* = 3 mice per genotype for FSK + IBMX) B cells after 24 h of unstimulated culture with or without forskolin + IBMX. (*C*) Percentage of viable GPR174 WT and KO B cells after 48 h of unstimulated culture (triplicate wells). (*D*) Percentage of viable sorted GPR174 WT and KO B cells after 24 h of unstimulated culture. *A*–*C* are representative of at least three experiments including male and female mice. Bar graphs represent mean ± SD. Statistical significance for *A*–*D* was determined by unpaired *t* test. ns, not significant; *P < 0.05; **P < 0.01; ***P < 0.001; ****P < 0.0001.

When murine B cell cultures were extended to 1 d, CD86 expression on WT B cells was reduced compared to that in 6 h of culture and the difference between WT and GPR174-deficient B cells was no longer evident, whereas G*α*s-deficient B cells continued to show decreased CD86 expression (Fig. 5*A*). In the 1-d cultures there was considerable B cell death (Fig. 5*B*). Notably, GPR174-deficient B cells underwent significantly less cell death than WT B cells, and this phenotype was more prominent with G*α*s-deficient B cells (Fig. 5*B*). Addition of forskolin and IBMX to the cultures led to a substantial further reduction in B cell viability and this effect was partially dependent on GPR174 and fully dependent on G*α*s (Fig. 5*B*). Although WT B cell cultures had few viable cells after 48 h of unstimulated incubation, GPR174 deficiency still conferred a protection against cell death (Fig. 5*C*). This viability effect was not dependent on the presence of other cells in the culture, as fluorescence-activated cell sorting (FACS)-purified B cells showed a similar viability difference to B cells from total splenocytes (Fig. 5*D*).

### GPR174-G*α*s Promotes NUR77 Expression In Vitro and In Vivo.

**Fig. 6.**
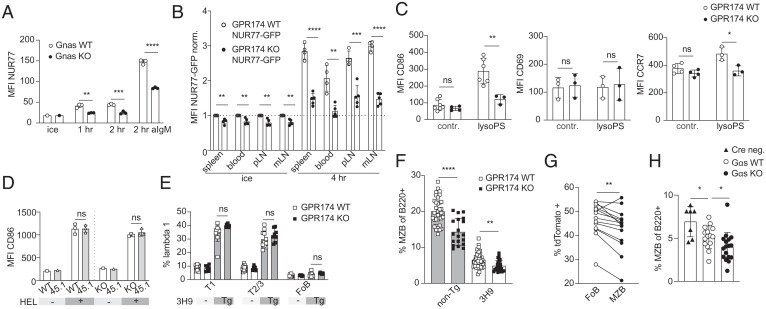
GPR174-G*α*s promotes NUR77 expression in vivo and influences MZ size. (*A*) MFI of intracellular NUR77 on splenic B cells from G*α*s WT or KO mice cultured for the indicated times with or without anti-IgM or maintained on ice (triplicate wells). (*B*) MFI of NUR77-GFP reporter on GPR174 WT (*n* = 4 mice) or KO (*n* = 5 mice) male follicular B cells from indicated tissues either immediately after harvesting (*Left*) or 4 h of unstimulated culture (*Right*). All values are normalized within each tissue to the WT sample that was maintained on ice. (*C*) MFI of CD86 (*Left*), CD69 (*Center*), and CCR7 (*Right*) on follicular B cells from draining popliteal lymph nodes of GPR174 WT or KO mice 4 to 6 h after footpad injection of 25 µg 18:1 lysoPS. Contr., contralateral (nondraining) lymph node. (*D*) MFI of CD86 on adoptively transferred GPR174 WT or KO MD4 (or CD45.1 congenic WT MD4) B cells 4 to 6 h after i.v. injection of 1 mg HEL or saline. (*E*) Percentage of $V_{\lambda }$1 positive splenic T1 transitional (T1, CD93^+^CD23^--^), T2/3, or follicular (FoB) B cells from GPR174 3H9 transgenic or nontransgenic mice (N ≥ 7 mice per genotype). (*F*) Percentage of MZ B cells (MZB, CD21/35^+^CD23^--^) of total splenic B220^+^ cells in GPR174 3H9 transgenic or nontransgenic mice (N ≥ 20 mice per genotype). (*G*) Percentage of tdTomato reporter positive of follicular or MZ B cells from *Gpr174^+∕--^* female mice (*n* = 14 mice). Lines connect percentage between compartments within the same mouse. (*H*) Percentage of MZB of splenic B220^+^ cells from *Gnas^+∕+^ CD19 Cre^+∕--^* (WT, N = 15), *Gnas ^fl∕fl^ CD19 Cre^+∕--^* (KO, *n* = 17), or *Cre^--^* control mice (*n* = 7). *A*, *C*–*F*, and *H* are representative of at least three experiments including both male and female mice. Statistical significance for *A*–*F* and *H* was determined by unpaired *t* test. Bar graphs represent mean ± SD. Statistical significance for *G* was determined by paired *t* test. ns, not significant; *P < 0.05; **P < 0.01; ***P < 0.001; ****P < 0.0001.

One of the differentially expressed genes in cultured WT B cells at both 1 and 4 h was *Nr4a1*, encoding NUR77, an early response gene that is well studied in lymphocytes for its induction via antigen receptor signaling (37, 38). To validate GPR174- and G*α*s-dependent induction of *Nr4a1* we performed intracellular staining of NUR77 in WT and G*α*s-deficient mice. NUR77 protein levels were spontaneously induced in WT but not G*α*s-deficient B cells within 1 h of culture in the absence of stimulation (Fig. 6*A*). While treatment with anti-IgM increased NUR77 expression in both WT and G*α*s-deficient B cells, expression in G*α*s-deficient B cells was not fully rescued. WT and G*α*s-deficient B cells showed comparable levels of NUR77 protein immediately ex vivo. However, B cells from WT and NUR77 KO mice also showed similar levels of NUR77 staining (*SI Appendix*, Fig. S5*A*), indicating that the antibody staining was not sufficient to detect the low levels of NUR77 that exist at the steady state in mature B cells.

NUR77-GFP mice show a high level of reporter in mature naive B cells due to the stability of GFP, despite rapid turnover of NUR77 in vivo (39, 40). Thus, we reasoned that the NUR77 reporter on a GPR174-deficient background might reveal subtle differences in NUR77 production or turnover. NUR77-GFP conferred a high level of GFP in follicular B cells compared to reporter negative mice, and B cells from WT NUR77-GFP mice had elevated GFP levels across multiple tissues compared to GPR174-deficient NUR77-GFP B cells (Fig. 6*B* and *SI Appendix*, Fig. S5*B*). Furthermore, WT NUR77-GFP B cells displayed more spontaneous GFP induction than GPR174-deficient NUR77-GFP B cells upon in vitro culture (Fig. 6*B* and *SI Appendix*, Fig. S5*B*). These observations provide evidence that GPR174 signaling influences gene expression in vivo under homeostatic conditions.

### Chemokines Do Not Alter GPR174-Dependent CD86 Induction.

GPR174 has been reported to bind and respond to CCR7 ligands CCL19 and CCL21 (3). At concentrations that promote the migration of CCR7-expressing cells, recombinant CCL19 and CCL21 had no effect on the CD86 up-regulation by WT B cells in vitro (*SI Appendix*, Fig. S5*C*). Prior work found reduced migration of *Gpr174 ^---∕Y ^*(male) B cells preactivated for 48 h with anti-IgM and anti-CD40 to CCL19 and CCL21. In the mice studied here we did not observe an effect of GPR174 deficiency on preactivated male B cell migration to CCL19 or CCL21 (*SI Appendix*, Fig. S5*D*). The prior work also reported that CCR7-transfected 293T cells bound to a histidine (His)-tagged form of CCL21 (3). CCL21 has a highly positively charged C terminus that can lead to binding to negatively charged proteoglycans (41). In contrast, CCL19 lacks this positively charged C terminus, so we used recombinant CCL19-Fc, a reagent previously used to study CCR7 expression (42), to test for binding to GPR174. Using transfected 293T cells that had high surface expression of the GPR174 epitope tag (OX56), we were unable to detect CCL19-Fc binding (*SI Appendix*, Fig. S5*E*). Expression of GPR174 fused at the C terminus with GFP also did not confer binding to CCL19-Fc (*SI Appendix*, Fig. S5*E*). In comparison, cells transfected with CCR7 showed a high level of CCL19-Fc binding as expected (*SI Appendix*, Fig. S5*E*). Similarly, CCL19-His bound to CCR7 but not GPR174-expressing 293T cells (*SI Appendix*, Fig. S5*F*).

Our RNA-sequencing result showed that cultured B cells undergo a GPR174- and G*α*s-dependent induction of *Ccr7* (Figs. 3*C* and 4*C*). In agreement with these data, surface levels of CCR7 increased to a greater extent in cultured WT versus GPR174-deficient B cells (*SI Appendix*, Fig. S5*G*). Unstimulated T cells increased CCR7 expression to a lesser extent after 6 h of culture and the level was not different in WT versus GPR174-deficient cells (*SI Appendix*, Fig. S5*G*). CCL19-Fc binding to B cells increased in accord with CCR7 levels (*SI Appendix*, Fig. S5*H*) and there was no binding of CCL19-Fc to freshly isolated or cultured CCR7-deficient B cells (*SI Appendix*, Fig. S5*H*). In support of the ability of G*α*s-cAMP to induce CCR7, stimulation with forskolin and IBMX increased CCR7 expression and CCL19-Fc binding in B cells (*SI Appendix*, Fig. S5 *G* and *H*). G*α*s signaling has previously been shown to induce CCR7 expression and function in monocytes (43).

### LysoPS Promotes B Cell CD86 Expression In Vivo.

In vitro studies have established that several forms of lysoPS (16:1, 18:0, and 18:1 in particular) can act as ligands for GPR174 (1, 2, 7, 8, 23, 24). Although we had not detected an effect of exogenous lysoPS on B cell GPR174 signaling in vitro, the rapid increase in lysoPS that occurred upon B cell culture (*SI Appendix*, Fig. S1*J*) led us to ask whether we could observe an effect of elevating lysoPS abundance in vivo. Mice were injected with lysoPS in the footpad and the draining popliteal lymph node was monitored 4 h later. LysoPS treatment led to up-regulation of CD86 but not CD69 in draining lymph node B cells, and not in contralateral lymph node B cells, in a GPR174-dependent manner (Fig. 6*C*). LysoPS also led to a detectable increase in B cell CCR7 expression (Fig. 6*C*) and NUR77-GFP reporter expression (*SI Appendix*, Fig. S5*I*). A similar experiment in conditional G*α*s-deficient mice showed that G*α*s was required for lysoPS-mediated induction of CD86 and CCR7 (*SI Appendix*, Fig. S5*J*).

To test for altered antigen-induced CD86 up-regulation in GPR174-deficient cells in vivo, B cells from GPR174-deficient mice carrying hen egg lysozyme (HEL)-specific transgenic IgM and IgD (MD4) were transferred into WT hosts. Four to six hours following HEL immunization there was comparable induction of CD86 on WT and GPR174-deficient MD4 B cells (Fig. 6*D*). These data indicate that under these in vivo conditions, BCR-signaling–induced CD86 does not depend on GPR174 signals. Consistent with these findings, G*α*s-deficient mice mounted a normal germinal center response to a T-dependent antigen (*SI Appendix*, Fig. S5*K*).

### GPR174 Influences Marginal Zone B Cell Compartment Size.

SNPs in GPR174 are associated in several GWAS with autoimmune disease (4–6), and NUR77 plays a role in the pruning of peripheral autoreactive B cells in a mouse model of lupus (44). V_H _3H9 (3H9) mice express a transgenic Ig heavy chain that, when paired with endogenous light chains, leads to double-stranded DNA (dsDNA)-reactive B cells that are deleted during development (45–47). The V*_λ_*1 light chain is the most autoreactive and deletion in 3H9 mice preserves tolerance. Given the coincidence of GPR174 expression in B cell development with the pruning of dsDNA-autoreactive B cells and a potential role of this receptor in human autoimmune disease, we asked whether loss of GPR174 influenced tolerance in this model. WT 3H9 and GPR174-deficient 3H9 mice showed similar levels of V*_λ_*1 light chain deletion from T1 transitional to mature follicular B cells (Fig. 6*E*). Thus, tolerance appears to be intact. However, in the course of this work we noted that the percentage of splenic MZ B cells, which is expanded in 3H9 transgenic mice, was decreased in GPR174-deficient 3H9 compared to WT 3H9 mice (Fig. 6*F*). An assessment of a large number of WT and GPR174-deficient mice showed that GPR174 deficiency led to a reduction in the MZ B cell compartment of polyclonal mice (Fig. 6*F*). This GPR174-dependent defect was cell intrinsic, as *Gpr174* heterozygous females, in which random X inactivation would yield ∼50% reporter-positive cells, had lower percentages of tdTomato-positive (GPR174-deficient) cells in the MZ versus the follicular B cell compartment (Fig. 6*G*). Testing the effect of G*α*s deficiency on the MZ compartment was complicated by the *Cd19-Cre* allele being a KO allele of CD19, since CD19 loss is associated with a reduction in the MZ (48). *Cd19-Cre* heterozygosity did lead to reduced MZ size but comparing *Gnas^+∕+^ Cd19-Cre^+∕--^* and *Gnas^fl∕fl^ Cd19-Cre^+∕--^* mice suggested that G*α*s was also required for maintenance of a MZ compartment of normal size (Fig. 6*H*).

## Discussion

In this work we reveal that a GPR174-dependent gene expression program is induced in cultured B cells. This program is dependent on G*α*s, providing evidence that GPR174 in B cells signals via G*α*s-containing heterotrimeric G proteins. Notable GPR174-G*α*s–induced genes are *Nr4a1*, *Cd86*, *Ccr7*, and phosphodiesterases while GPR174-G*α*s–repressed genes include ITIM-containing receptors *Cd22* and *Btla*. Importantly, GPR174-G*α*s signaling reduces B cell viability in culture. We provide in vivo evidence that GPR174 can signal via this pathway through the finding of reduced NUR77-GFP reporter expression in mice lacking the receptor and the demonstration that lysoPS treatment increases CD86, NUR77, and CCR7 in lymph node B cells. GPR174 and G*α*s also contribute to the induction or maintenance of the MZ B cell compartment. These findings complement earlier observations on GPR174 function in T cells (2, 7, 8) in advancing our understanding of how variants in the GPR174 locus could contribute to human autoimmune disease (4–6). They also provide insights that may enable the development of improved approaches for studying B cells in culture.

The cAMP inducibility of CD86 was observed in multiple early studies with mouse and human cells (13–18, 49, 50). However, most subsequent work highlighting the ability of the BCR and other B cell-activating receptors to induce CD86 has been consistent with the NFkB inducibility of this costimulatory molecule (11, 51, 52). The BCR is not thought to engage G*α*s signaling, and we speculate that the weaker up-regulation of CD86 after in vitro BCR stimulation in GPR174- and G*α*s-deficient B cells is reflective of the CD86 induction in WT B cells representing the additive effect of the spontaneous GPR174-G*α*s (cAMP) signal and the BCR-induced (NFkB) signal. The physiological context in which cAMP contributes to CD86 induction in B cells remains to be defined although the *β*2-adrenergic receptor and prostacyclin receptor PTGIR have also been implicated in this process (50, 53).

LysoPS can be generated from phosphatidylserine (PS) by intracellular and extracellular enzymes (54, 55), and lysoPS abundance in tissue increases under inflammatory conditions (55, 56). A surprising observation in our work was that GPR174-dependent CD86 induction was unaffected by the addition of lysoPS to the cultures. However, lymphocytes can generate lysoPS (8, 57, 58) and our findings indicate that lysoPS abundance in B cells increases within minutes of in vitro incubation. We speculate that the lack of activity of exogenously added lysoPS reflects efficient occupancy of the receptor by lysoPS in an autocrine manner. However, our mouse lysoPS treatment data suggest that under homeostatic conditions in vivo the receptor is not fully occupied and exogenous lysoPS can engage the receptor and induce a CD86-containing gene expression program.

It is important to consider how our findings relate to evidence that GPR174 is a receptor for CCL19 and CCL21 (3). Our inability to detect an influence of CCL19 or CCL21 on GPR174-dependent CD86 induction is consistent with our inability to detect CCL19 binding to GPR174. Further work will be needed to resolve the discrepancy between Zhao et al. (3), detecting CCL21-His binding, and our inability to detect CCL19-His binding to GPR174-transfected HEK293T cells. Given our in vitro and in vivo findings that CCR7 can be up-regulated in naive B cells in a GPR174- and G*α*s-dependent manner, we speculate that under some conditions GPR174 promotes CCL19 and CCL21 responses by increasing CCR7 expression. Zhao et al. (3) provided biochemical evidence for GPR174 coupling to G*α*i and G*α*13 in anti-IgM plus anti-CD40–activated B cells (3). Although we have not performed biochemical studies, we believe the very close phenocopy observed in our in vitro and in vivo experiments between GPR174-deficient and G*α*s-deficient B cells provides strong evidence that GPR174 signals via G*α*s-containing heterotrimeric G proteins in naive B cells. A profiling study of GPCR-coupling capabilities reported that GPR174 strongly engaged G*α*s but could stimulate G*α*13 and G*α*i more weakly (59). Zhao et al. (3) noted as an unpublished observation that when CCR7 is ablated in activated B cells, GPR174-G*α*i coupling and GPR174-mediated migration are markedly reduced. Further studies will be needed to discern whether GPR174 undergoes a change in G-protein coupling during B cell activation and whether this involves GPR174 interaction with CCR7.

cAMP signaling in activated B cells has previously been associated with reduced viability (60). The basis for GPR174 and G*α*s signaling reducing B cell viability in vitro may reflect induction of several genes. For example, NUR77 restrains B cell survival (61). *Cables1* (Cdk5 and Abl enzyme substrate 1), one of the most strongly GPR174-dependent genes, can promote apoptosis (62). A growth-repressive influence of G*α*s on B cells is suggested by the finding of *GNAS* mutations in human diffuse large B cell lymphomas (63). Based on studies on T cells and innate immune cells, cAMP signaling is generally considered to be immune suppressive (64). Indeed, G*α*s-coupled adenosine receptors are targets of therapeutic antagonists that are being developed to increase tumor-specific T cell responses. However, studies in G*α*s-deficient T cells showed a positive role for G*α*s in promoting Th17 and Th1 cell differentiation and inflammatory function (65). When the activity of the GPR174-G*α*s pathway in promoting costimulatory and reducing ITIM molecule expression in B cells is taken together with its activity in reducing their viability, we suggest it may have both stimulatory and suppressive influences on B cells, helping fine-tune certain responses.

Our findings demonstrate that *Nr4a1* is a G*α*s-inducible gene in B cells. Consistent with these data, *Nr4a1* is a CREB target gene in adrenal medulla-derived PC12 cells (66) and hepatocytes (67). NUR77 has a role in restricting the survival of self-reactive peripheral B cells and restraining B cell responses to antigen in the absence of signal 2 (44, 61). The latter activity involved partially redundant roles of *Nr4a* family members. Reduced expression of *Nr4a1* and other *Nr4a* family members in GPR174-deficient B cells may lead to less effective control of self-reactive B cells. Whether the *Cd86* gene is directly induced by CREB is less clear although the GPR174-dependent up-regulation of transcripts within 1 h of incubation suggests direct induction by a cAMP-responsive factor. A study of human cells provided evidence for functional CREB-binding sites in the *CD86* promoter (68) but these sites are not well conserved in the mouse *Cd86* promoter (32). However, CREB can bind to many noncanonical sites (69, 70). Future pCREB chromatin-immunoprecipitation sequencing (ChIPseq) studies in B cells will help identify the full set of genes that are directly targeted by G*α*s signaling.

The MZ B cell compartment has unique signaling requirements compared to follicular B cells, including a dependence on BCR, CD19, and Notch signaling (48, 71). MZ B cells are also dependent on G*α*i- and G*α*12/13-coupled GPCRs for their positioning and homeostasis (72, 73). How GPR174 and G*α*s signals integrate with these other inputs to promote the normal accumulation of MZ B cells is not yet clear. A previous study examining a different GPR174-deficient mouse line, also on the C57BL/6 background, suggested that GPR174 deficiency was associated with a marked increase in the MZ compartment (74). The basis for this discrepancy in findings is unclear but there is some evidence that the microbiome, which is likely different between facilities, can influence the MZ compartment (75, 76). Although our GPR174-deficient mice were generated by gene targeting in 129-background ES cells followed by extensive backcrossing to C57BL/6J, our findings are unlikely to be a consequence of linked gene variants because we obtained similar results in G*α*s-deficient mice.

In summary, we provide evidence that GPR174 is the dominant G*α*s-coupled GPCR in naive B cells. We find that B cells undergo substantial gene expression change during the early period of in vitro culture and that a considerable amount of this change is GPR174 and G*α*s dependent. In vivo, GPR174 appears capable of engaging a similar pathway and we propose that the receptor helps tune B cell responses based on the tissue microenvironment and state of inflammation. Altered activity of the GPR174 signaling pathway in B cells may contribute to development of Graves’, Addison’s, and possibly other autoimmune diseases.

## Materials and Methods

### Mice.

All mice were bred internally. GPR174-deficient tdTomato reporter mice were previously described (2). Gnas floxed mice (25) (MGI: 3043361) were provided by Guo Huang, University of California, San Francisco, CA, and were backcrossed 10 times to C57BL/6J. CD19-Cre mice [B6.129P2(C)-Cd19tm1(cre)Cgn/J] were from an internal colony. The 3H9 and NUR77 reporter mice were obtained from J. Zikherman, University of California, San Francisco, CA. CD45.1 B6 (B6.SJL-PtprcaPepcb/BoyCrCrl) mice used for chimera recipients and cell transfer experiments were bred internally from founders ordered from JAX. All mice were analyzed between 8 and 20 wk of age except for marginal zone B cell analyses, in which mice were analyzed at 10 to 20 wk of age. Mice were cocaged with littermates for all experiments. All data are representative of male and female mice unless otherwise noted. Animals were housed in a specific pathogen-free environment in the Laboratory Animal Research Center at UCSF and all experiments conformed to ethical principles and guidelines approved by the UCSF Institutional Animal Care and Use Committee.

### Bone Marrow Chimeras.

CD45.1 B6 mice were lethally irradiated with 1,100 rads gamma irradiation (split dose separated by 3 h) and then i.v. injected with relevant BM cells under isoflurane anesthesia. Chimeras were analyzed after 10 to 12 wk of reconstitution.

### Retroviral Constructs and Transductions.

Murine PKA-AB sequence was obtained from S. McKnight, University of Washington School of Medicine, Seattle, WA, and cloned into the MSCV2.2 retroviral vector followed by an internal ribosome entry site (IRES) and GFP as an expression marker. Retrovirus was generated by transfecting the PLAT-E packaging cell line with 10 µg plasmid DNA and 25 µL Lipofectamine 2000 (Fischer). Bone marrow donors were injected with 3 mg of 5-fluorouracil (Sigma) and bone marrow was collected 4 d later and cultured in Dulbecco’s modified Eagle medium (DMEM) containing 15% (vol/vol) FBS, antibiotics (penicillin [50 IU/mL] and streptomycin [50 µg/mL]; Cellgro), and 10 mM Hepes, pH 7.2 (Cellgro), supplemented with IL-3, IL-6, and stem cell factor (at concentrations of 20, 50, and 100 ng/mL, respectively; PeproTech). Cells were “spin infected” twice at days 1 and 2 of culture at 2,400 rpm and 37 ^∘^C with viral supernatants and were transferred into irradiated recipients on day 3.

### In Vitro B Cell Culture.

Spleens were isolated and sterilely mashed through a 100-µm filter on ice in cold MACS (PBS with 2% FBS and 1 mM EDTA). Spleens were washed and diluted in complete RPMI 1640 (containing 10% FBS, 10 mM Hepes, 55 µM 2-mercaptoethanol, 2 mM glutamine, and 50 IU penicillin/streptomycin). Cells were plated (5 × 10^5^) in 96-well flat-bottom plates. Plates were incubated at 37 ^∘^C with 5% CO_2_ for the indicated times and then collected, washed, and stained in 96-well round-bottom plates. Chemicals and stimulations were added to cultures at the following concentrations: 10 µM forskolin (Cayman Chemicals) and 100 µM IBMX (Sigma), 10 µg ⋅mL^--1^ anti-IgM (Jackson ImmunoResearch), 10 µM 18:1 LysoPS (Avanti Polar Lipids), 100 ng ⋅mL^--1^ CXCL12 (Peprotech), 500 ng ⋅mL^--1^ CCL19 (R&D Systems), 500 ng ⋅mL^--1^ CCL21 (R&D Systems), 0.5 µg ⋅mL^--1^ LEAF-purified anti-CD180 (Fisher), 10 µM H89 (Fisher), 1 µM Ibrutinib (Selleck Chemicals), 1 µM BAY61-3606, and 20 µM PP2.

### Flow Cytometry.

Cells were stained for 20 min on ice in MACS buffer (2% FCS in PBS with 1 mM EDTA) at 0.5 to 1 × 10^6^ cells per well in 96-well round-bottom plates unless otherwise specified. All mAbs were purchased from BioLegend unless otherwise indicated. The following monoclonal antibodies were used: TCR*β*–BV421, TCR*β*–PerCP-Cy5.5 (Tonbo), CD23–PE-Cy7, CD23–Alexa Fluor 488, CD86–Alexa Fluor 647, CD86–PerCP-Cy5.5, CD21/35–Pacific Blue, B220–BV785, CD69–FITC (BD), CD93–APC (eBioscience), CD4–BV605, CD45.2–PE, CD45.1–BV605, lambda-1 light chain–biotin (BD) (followed by streptavidin–BV605), CD19–PE, and CCR7–biotin (followed by streptavidin–BV421). CCR7-biotin was stained for 1 h at room temperature followed by streptavidin and other surface markers on ice. Dead cells were excluded using Fixable Viability Dye eFluor780 (eBioscience no. 65-0865-18). For time-course experiments all samples were plated simultaneously, collected and kept on ice, and stained in parallel. Viability for 24- to 48-h cultures was determined by pregating on B220^+^ TCR*β*^--^. All samples were run on a BD LSRii at 5,000 to 10,000 events per second. Flow cytometry data were analyzed using FlowJo (v10.8.0).

### Cell Sorting and RNA Sequencing.

Follicular B cells were sorted from spleens of male mice into cold MACS buffer using a BD FACSAria II. Sort purity was greater than 99% for *Gpr174^+∕Y ^*and *Gpr174^--∕Y ^*B cells and 95 to 99% for *Gnas^+∕+^ Cd19 Cre^+∕--^* and *Gnas^fl∕fl^ Cd19 Cre^+∕--^* B cells. A total of 5 × 10^6^ cells were either frozen immediately postsort or following 1 or 4 h of culture in complete RPMI. The GPR174 4-h experiment was prepared for sequencing using Ovation RNA-seq System V2 from Nugen, the KAPA Hyper prep labeling kit, and the NEXTflex DNA barcodes Adapter kit from Bioo Scientific, and 50 bp single end was run on HiSeq2500 at the UCSF Institute for Human Genetics. The GPR174 1-h and G*α*s 4-h experiments were prepared for sequencing using the QuantSeq 3′ kit from Lexogen and 50 bp single end was run on HiSeq4000 at the UCSF Center for Advanced Technology. Sequences were aligned to the mm10 genome with STAR and mapped reads of each gene were counted with HTseq. DESeq2 was used for the gene differential expression analysis and with GSEA software. The heatmap was generated with pheatmap. Volcano plots were generated with EnhancedVolcano.

### In Vitro B and T Cell Proliferation.

Follicular B cells were purified from GPR174 WT and KO spleens by depletion using biotinylated antibodies (anti-CD43, anti-TER119, anti-CD11c, anti-TCR*β*, anti-CD4, and anti-CD8) and streptavidin-conjugated beads (EasySep Streptavidin RapidSpheres) to greater than 95% purity. CD4 T cells were purified using anti-TER119, anti-B220, anti-CD11c, and anti-CD8 and streptavidin-conjugated beads to greater than 95% purity. B/T cells were labeled with CellTrace Violet (Life Tech) according to the manufacturer’s protocol. The 24-well plates were coated with anti-CD3 (2 µg ⋅mL^--1^) and anti-CD28 (2 µg ⋅mL^--1^) (both LEAF purified from BioLegend) in PBS for 3 h at 37 ^∘^C and T cells were plated (4 × 10^5^ cells per well) in complete RPMI 1640 with or without 10 µM 18:1 LysoPS (Avanti Polar Lipids). B cells (4 × 10^5^ cells per well) were plated in 24-well plates with LPS (10 µg ⋅mL^--1^ 0111:B4 from *Escherichia coli*; Sigma) and with or without 18:1 LysoPS. Proliferation of viable cells was determined after 4 d.

### Intracellular NUR77 Staining.

Splenocytes were isolated and cultured as indicated in the figure legends except for ice samples, which were kept on ice until all samples were collected and then stained concurrently. Cells were collected and washed with cold MACS buffer (2% FCS in PBS with 1 mM EDTA) in 96-well round-bottom plates (1 × 10^6^ cells per well). Cells were stained with Fixable Viability Dye eFluor780 for 20 min on ice, washed twice with MACS, and then resuspended in 100 µL 2% PFA and incubated for 10 min at room temperature. Cells were washed twice with MACS and the pellet was dislodged by vigorously tapping the plate; then 150 µL of ice-cold methanol was added dropwise to the wells. The plates were then kept at –20 ^∘^C for 45 min and then washed and rehydrated for 5 min with MACS. Following another wash, cells were stained with NUR77-PE (eBiosciences no. 12-5965-80), B220–BV785, TCR*β*–BV421, CD23–PE-Cy7, and the relevant surface markers for 1 h at room temperature and then washed twice, resuspended in MACS, and collected on a BD LSRii.

### Migration Assays.

B cells were enriched from spleens of cocaged littermate males by depletion using biotinylated antibodies (anti-CD43, anti-TER119, anti-CD11c, anti-TCR*β*, anti-CD4, and anti-CD8) and streptavidin-conjugated beads (EasySep Streptavidin RapidSpheres). B cells were activated with 10 µg ⋅mL^--1^ F(ab′)2 goat anti-mouse IgM (Jackson Immunoresearch) and 10 µg ⋅mL^--1^ anti-CD40 (clone FGK4.5; Bio X Cell) in complete RPMI for 60 h. B cells were collected, washed in prewarmed migration medium (RPMI containing 0.5% fatty acid-free BSA, 10 mM Hepes, and 50 IU penicillin/streptomycin), and rested in migration medium at 37 ^∘^C for 1 h. B cells (200 µL, 1 × 10^6^cells) were then added to transwells (5-µm pore; Corning Costar no. 93421) with the indicated chemokines in migration media (600 µL) in the bottom chamber and allowed to migrate for 3 h at 37 ^∘^C and 5% CO_2_. Migrated cells were then collected from the bottom chamber, stained for viable B220^+^ cells, and collected on a BD LSR II cytometer for a fixed time under constant flow rate and normalized to an “input” well.

### CCL19 Binding Assay.

HEK 293T cells were seeded in six-well plates with DMEM containing 10% FBS, 10 mM Hepes, 2 mM glutamine, and 50 IU penicillin/streptomycin and grown to 75% confluency and then transfected with the plasmids indicated in the figure legends in Lipofectamine2000 and Optimem (Life Technologies). One day following transfection, cells were dislodged and 5 × 10^5^ cells were washed in 96-well round-bottom plates. Cells were then resuspended and incubated with 1% Fc block (Bio X Cell) in MACS buffer (2% FCS in PBS with 1 mM EDTA) for 15 min on ice, and then CCL19-huFc (42) or CCL19-His (Cell Sciences CRM520A) was added without washing and incubated for 30 min on ice. Cells were washed twice with MACS and then stained with anti-huFc-PE (1:50; Jackson Immunoresearch 109-116-098) or anti-His-biotin (1:10; Miltenyi 130-099-423) for 20 min on ice. The secondary was preadsorbed with 2% each of normal rat serum and normal mouse serum. The His-biotin reagent was detected with streptavidin-BV421 (1:200; Biolegend 405226). Cells were washed twice, stained with Live/Dead and antibodies to identify vector-positive cells (with 1% normal rat serum and 1% normal mouse serum), and then washed twice and analyzed on a BD LSRii. Staining for detection of CCL19 binding on freshly isolated or 6-h cultured splenocytes was performed as for 293T cells using anti-huFc-AF488 (1:50; Jackson Immunoresearch 709-546-098) to detect the CCL19-huFc reagent.

### Immunization.

Mice were immunized intraperitoneally with 50 µg NP32-KLH and 1 µg LPS (0111:B4 from *E. coli*; Sigma) in 2% Alyhydrogel alum adjuvant (Invivogen) and spleens analyzed 14 d later.

### LysoPS Injections.

Mice were anesthetized using isofluorane and injected in the right footpad with 25 µg 18:1 lysoPS (Avanti Polar Lipids) or an equivalent volume of diluted solvent (methanol) in the contralateral footpad. Mice were euthanized 4 to 6 h later and popliteal lymph nodes harvested for flow cytometric analysis.

### Cell Culture and Sample Culture Preparation for LC-MS/MS.

Murine splenic B cells were purified by depletion using biotinylated antibodies (anti-CD43, anti-TER119, anti-CD11c, anti-TCR*β*, anti-CD4, and anti-CD8) and streptavidin-conjugated beads (EasySep Streptavidin RapidSpheres) to greater than 95% purity and resuspended at 5 × 10^7^ cells per milliliter in cold PBS. B cells (100 µL) were added to Eppendorfs containing 900 µL prewarmed PBS and incubated for the indicated times in a 37 ^∘^C heat block (or 900 µL cold PBS for ice conditions). Cells were pelleted at 400 relative centrifugal force (RCF) × 10 min at 4 ^∘^C, and the pellets frozen in liquid nitrogen. Samples were kept at –80 ^∘^C for a maximum of 12 h prior to sample preparation. Cell pellets were resuspended in 100 µL ice-cold methanol containing 0.1% formic acid and 10 nM 17:1 internal standard and homogenized using a Precellys 24 homogenizer with a Cryolys cooling unit (Bertin Technologies) and Precellys 0.5-mL soft tissue homogenizing tubes (Cayman Chemicals item 10428). Homogenization was conducted by two cycles for 15 s at 5,800 rpm (with a 30-s break) at a temperature lower than 4 ^∘^C. After sample homogenization, 70 µL lysate was recovered and spun at 16,000 RCF and 40 µL of supernatant was transferred to glass vials with 9-mm autosampler inserts and immediately used for LC-MS/MS analysis.

### LC-MS/MS.

Samples (15 µL volume) were injected into a Shimadzu Nexera HPLC system equipped with a binary pump and a SIL-30AC autosampler. Separation was achieved on a Synergi 4-µm Polar-RP 80Å(50 mm, 2 µm; Phenomenex). A gradient separation was used consisting of mobile phase A (H_2_O + 0.1% formic acid) and mobile phase B (acetonitrile + 0.1% formic acid) delivered at a flow rate of 1 mL/min. Mobile phase B was used at 30% from 0 to 1.0 min and then was increased linearly to 60% B from 1.0 to 6.0 min, followed by a quick ramp within 0.5 min to 95% B, which was maintained at 95% B for 2.5 min. After a quick ramp, within 0.5 min to 40% mobile phase B, it was maintained at 40% for another 0.5 min until the end of the analysis.

Mass spectrometric detection was performed using an AB SCIEX QTRAP 6500 operated in multiple-reaction monitoring (MRM) mode via the negative electrospray ionization interface using the transitions (*m/z*) 524 →153, 522 →153, and 508 →153 for 18:0 LysoPS, 18:1 LysoPS, and 17:1 LysoPS (as an internal standard), respectively. The ion source temperature was maintained at 400 ^∘^C. The spraying needle voltage was set at –4.5 kV. Curtain gas, collision gas (CAD), gas 1, and gas 2 were set at 20, medium, 40, and 40, respectively. The entrance potential (EP) was set at –10 V. The declustering potential and collision cell exit potential (CXE) were –150 and –19 V, respectively. Collision energy (CE) was –38 eV for all compounds. Data acquisition and quantitative processing were accomplished using the AB Sciex version 1.6.2 software and R to determine peak areas. Internal standard (17:1) peak areas were determined to have <5% variation across samples within each experiment, so 18:0 and 18:1 peak areas were used to determine sample concentration. Standard curves (18:0 and 18:1 lysoPS) contained at least two points below and above the range of sample values and an r value >0.99.

### Statistical Analyses.

Data were analyzed using a paired or unpaired Student’s *t* test as appropriate. Prism version 8 (GraphPad Software) was used for all statistical analyses and to generate plots. Each experiment was repeated at least three times, unless otherwise indicated in the figure legends. All error bars represent SDs.

## Supplementary Material

Supplementary File

Supplementary File

Supplementary File

Supplementary File

## Data Availability

All study data are included in this article and/or *SI Appendix*. RNAseq data have been deposited in Gene Expression Omnibus (GEO) (GSE200800) (31).
