# Supplementary material for: GPR174 signals via Gαs to control a CD86-containing gene expression program in B cells
Source: Proc Natl Acad Sci U S A. 2022 May 31;119(23):e2201794119. doi: 10.1073/pnas.2201794119 (PMC9191659; doi:10.1073/pnas.2201794119)
Supplement: Supplementary File [file pnas.2201794119.sapp.pdf]

PNAS
www.pnas.org

Supplementary Information for

GPR174 signals via Gαs to control a CD86-containing gene expression program in B cells

Elise W. Wolf, Zachary Howard, Lihui Duan, Hanson Tam, Ying Xu, Jason G. Cyster

Jason Cyster
E-mail: jason.cyster@ucsf.edu

This PDF file includes:

Figs. S1 to S5
Legends for Dataset S1 to S3

Other supplementary materials for this manuscript include the following:

Dataset S1 Dataset S2 Dataset S3

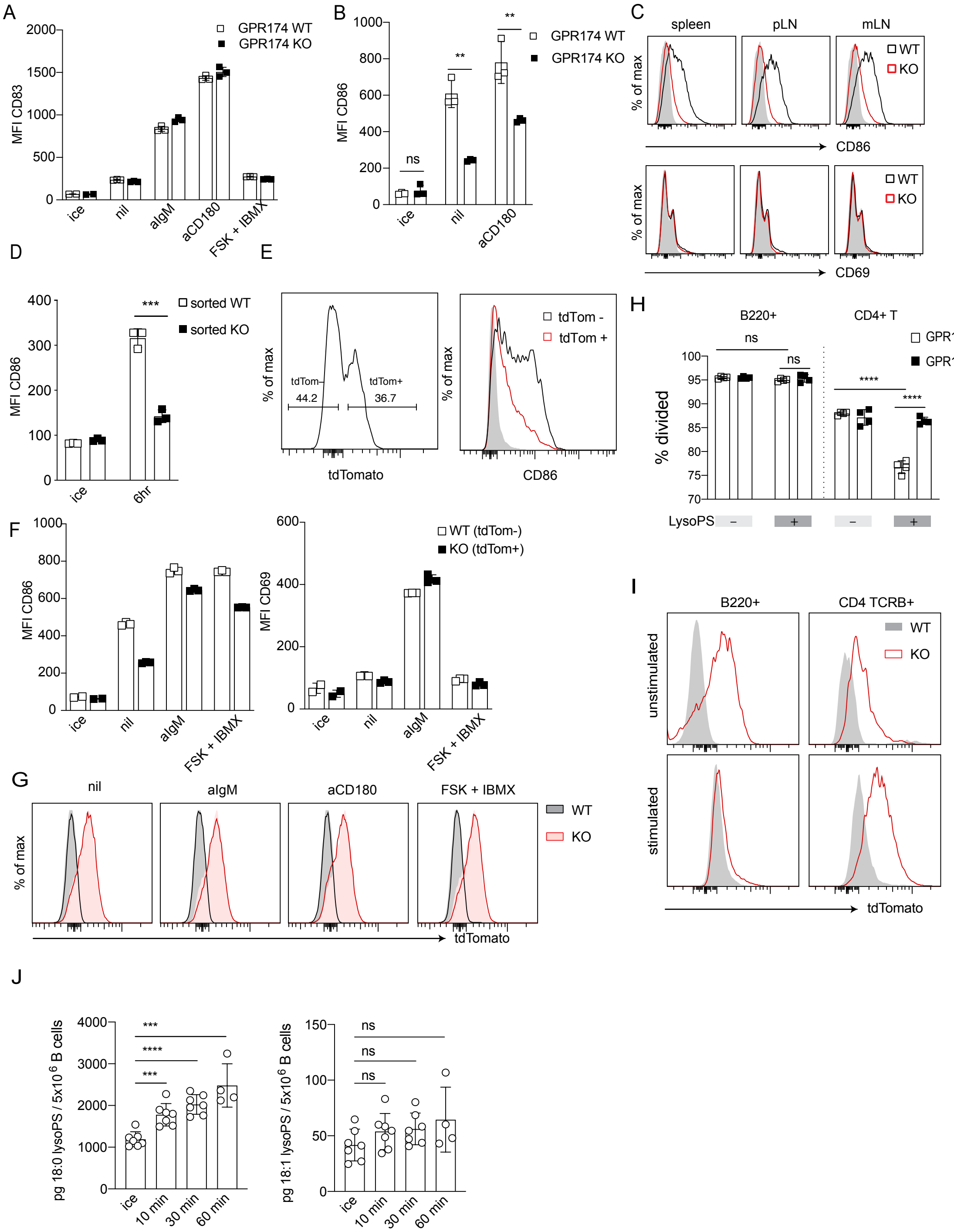

Fig. S1. GPR174 influences CD86 expression in B cells (A) MFI of CD83 on B cells from GPR174 WT or KO mice cultured for 6 hours with the indicated treatment or maintained on ice (triplicate wells). (B) MFI of CD86 on splenic follicular B cells from indicated genotypes cultured for 6 hours with or without anti-CD180 (0.5 μg ml^{-1}) or maintained on ice (N=3 mice per genotype). (C) Representative histograms of CD86 and CD69 expression on GPR174 WT or KO B cells from the indicated tissues after 6 hours of unstimulated culture, relative to cells maintained on ice (gray). (D) MFI of CD86 on sorted B cells immediately post-sort (ice) or after 6 hours of unstimulated culture (N=3 mice per genotype). (E) Representative gating of tdTomato reporter (left) and histogram of CD86 expression (right) of reporter negative or positive B cells from spleens of $Gpr174^{+/-}$ female mice after 6 hours of unstimulated culture, relative to cells maintained on ice (gray). (F) MFI of CD86 (left) or CD69 (right) on tdTomato reporter negative or positive B cells from $Gpr174^{+/-}$ female mice after 6 hours of culture with or without anti-IgM or forskolin + IBMX or maintained on ice (N=3 mice per genotype). (G) Representative histograms of tdTomato reporter expression on GPR174 WT or KO B cells immediately after isolation (solid) versus 6 hours of culture (lines) with the indicated stimulation. (H) Percent of viable $B220^{+}$ or $CD4^{+}$ T cells divided after 4 days in culture with or without 18:1 lysoPS (10 μM). Cells from GPR174 WT or KO spleens were enriched by depletion to >95% purity, CTV labeled, and stimulated with lipopolysaccharide (10 μg ml^{-1}) from E. coli (B cells) or plate-bound anti-CD3 (2 μg ml^{-1}) and anti-CD28 (2 μg ml^{-1}) (T cells). (I) Representative histograms of tdTomato reporter expression on cells from (H). (J) Amount of 18:0 (left) and 18:1 (right) lysoPS detected by LC-MS/MS in 5 x 10^{6} purified B cells cultured for the indicated amounts of time. (A, B, F, and J) are representative of at least three experiments. Statistical significance for (B, D, H, J) determined by unpaired T-test. ns = not significant; *p < 0.05; **p < 0.01; ***p < 0.001; ****p < 0.0001.

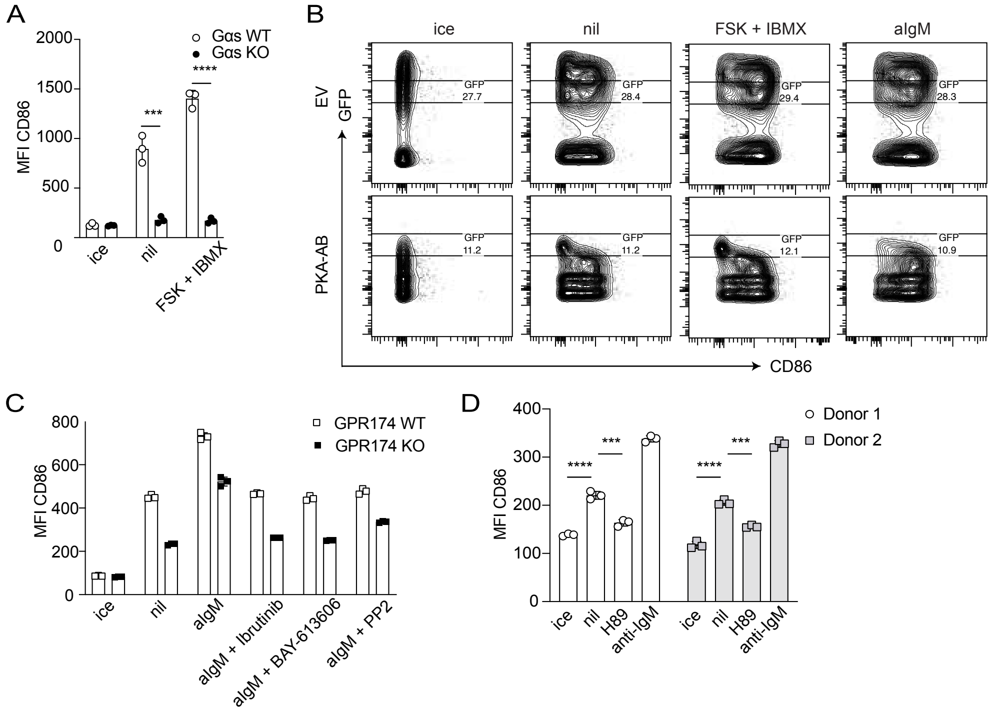

Fig. S2. CD86 induction is dependent on Gαs and PKA (A) MFI of CD86 on B cells from Gαs WT or KO mice after 6 hours of culture with or without forskolin + IBMX or maintained on ice (N=3 mice per genotype). (B) Representative plots of vector GFP and CD86 expression on B cells from chimeras transduced with EV GFP or PKA-AB GFP and cultured with or without anti-IgM or forskolin + IBMX or maintained on ice. (C) MFI of CD86 on B cells treated with or without anti-IgM, Ibrutinib (1 µM), BAY61-3606 (1 µM), or PP2 (20 µM) or maintained on ice (triplicate wells). (D) MFI of CD86 on $CD19^+$ cells from PBMCs of two human donors after 6 hours of culture under the indicated conditions or maintained on ice. (A and C) are representative of at least three experiments. Statistical significance for (A, D) determined by unpaired T-test. ns = not significant; *$p < 0.05$; **$p < 0.01$; ***$p < 0.001$; ****$p < 0.0001$.

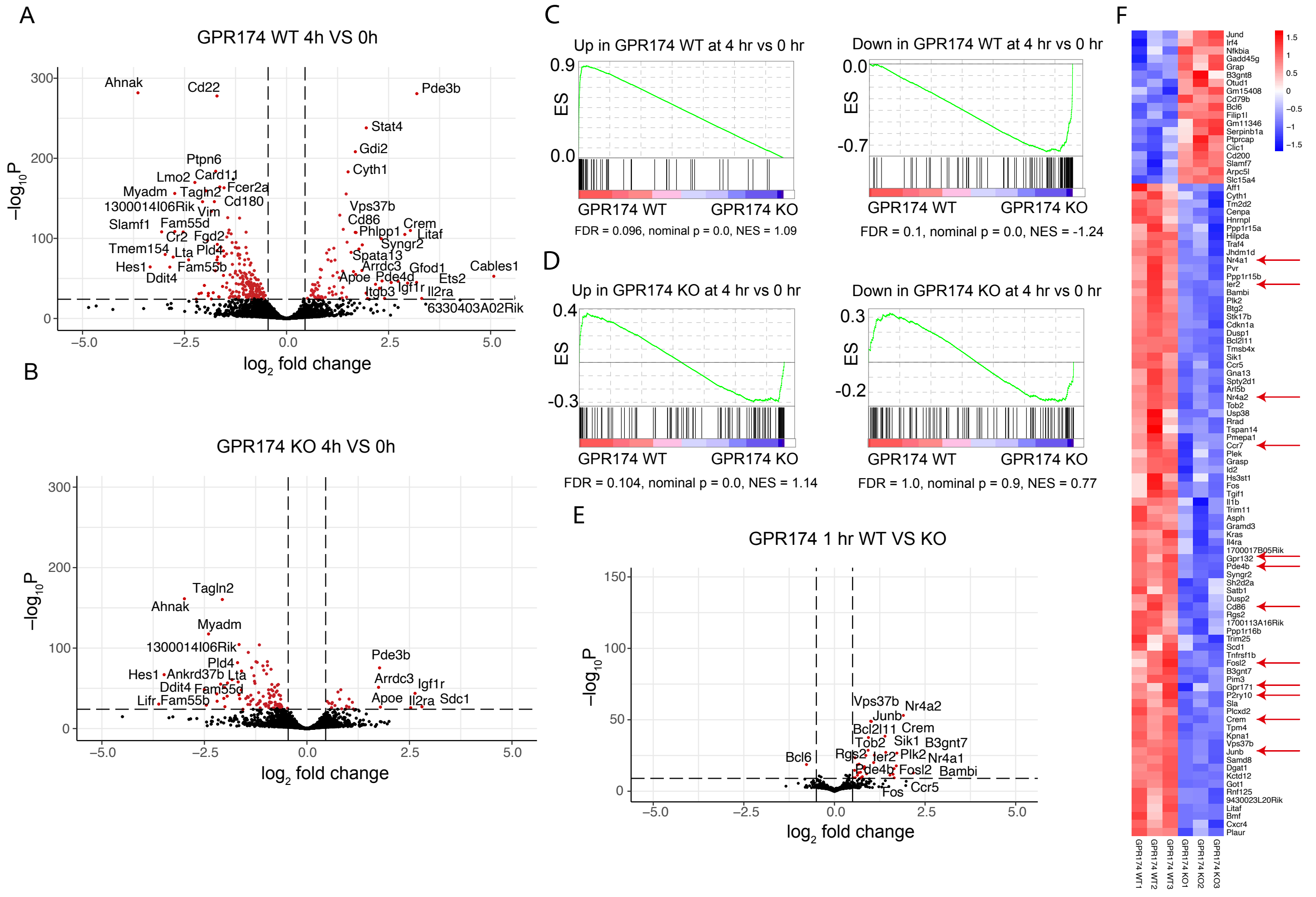

Fig. S3. GPR174 influences gene expression in B cells (A, B) Volcano plots showing differential gene expression at 4 versus 0 hours in GPR174 WT (A) or KO (B) B cells. (C) GSEA plots of the top 100 DEGs that increased (left) or decreased (right) in GPR174 WT B cells during culture compared to GPR174 WT versus KO after 4 hr of culture. (D) GSEA plots of the top 100 DEGs that increased (left) or decreased (right) in GPR174-deficient B cells during culture compared to GPR174 WT versus KO after 4 hr of culture. (E) Volcano plot showing differential gene expression in GPR174 WT versus KO B cells after 1 hour of culture. (F) Heatmap of top 100 DEGs between GPR174 WT and KO B cells after 1 hour of culture. Red arrows indicate genes referenced in the text. For volcano plots, dashed lines indicate fold-change and p-value cutoffs for highlighted DEGs.

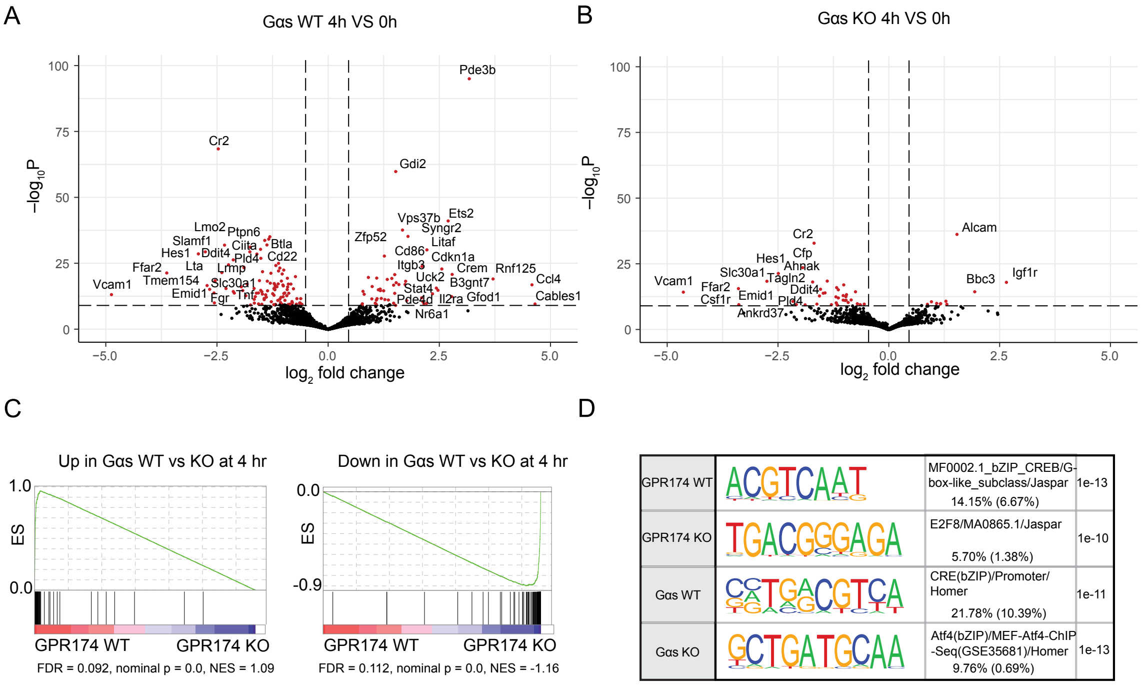

E

Gene		chol. synth
Alcam	Activated leukocyte cell adhesion molecule	no
Cyp51	Sterol 14-demethylase	yes
Fdft1	Farnesyl-diphosphate farnesyltransferase 1	yes
Fdps	Farnesyl diphosphate synthase	yes
Hmgcs1	Hydroxymethylglutaryl-CoA synthase	yes
Igf1r	Insulin-like growth factor 1 receptor	no
Ldlr	Low density lipoprotein receptor	yes
Lilrb4	Leukocyte immunoglobulin like receptor B4)	no
Mcoln2	Mucolipin-2	no
Pnpla7	Patatin-like phospholipase domain-containing protein 7	no
Sc4mol	Methylsterol monooxygenase 1	yes
Sqle	Squalene epoxidase	yes

Fig. S4. Gαs-deficient B cells phenocopy GPR174-deficient gene expression changes (A, B) Volcano plots showing differential gene expression at 4 versus 0 hours in Gαs WT (A) or KO (B) B cells. Dashed lines indicate fold-change and p-value cutoffs for highlighted DEGs. (C) GSEA plots of the top 100 DEGs increased (left) or decreased (right) in Gαs WT versus KO B cells compared to GPR174 WT and KO B cells that were cultured for 4 hours. (D) Top de novo motif logos (Homer) for DEGs upregulated during culture in B cells of the indicated genotype, with percent of DEGs containing the motif and, in parentheses, percent background. (E) Overlap of genes that increase in GPR174-deficient and Gαs-deficient B cells during culture more than in WT cells and their involvement in cholesterol biosynthesis.

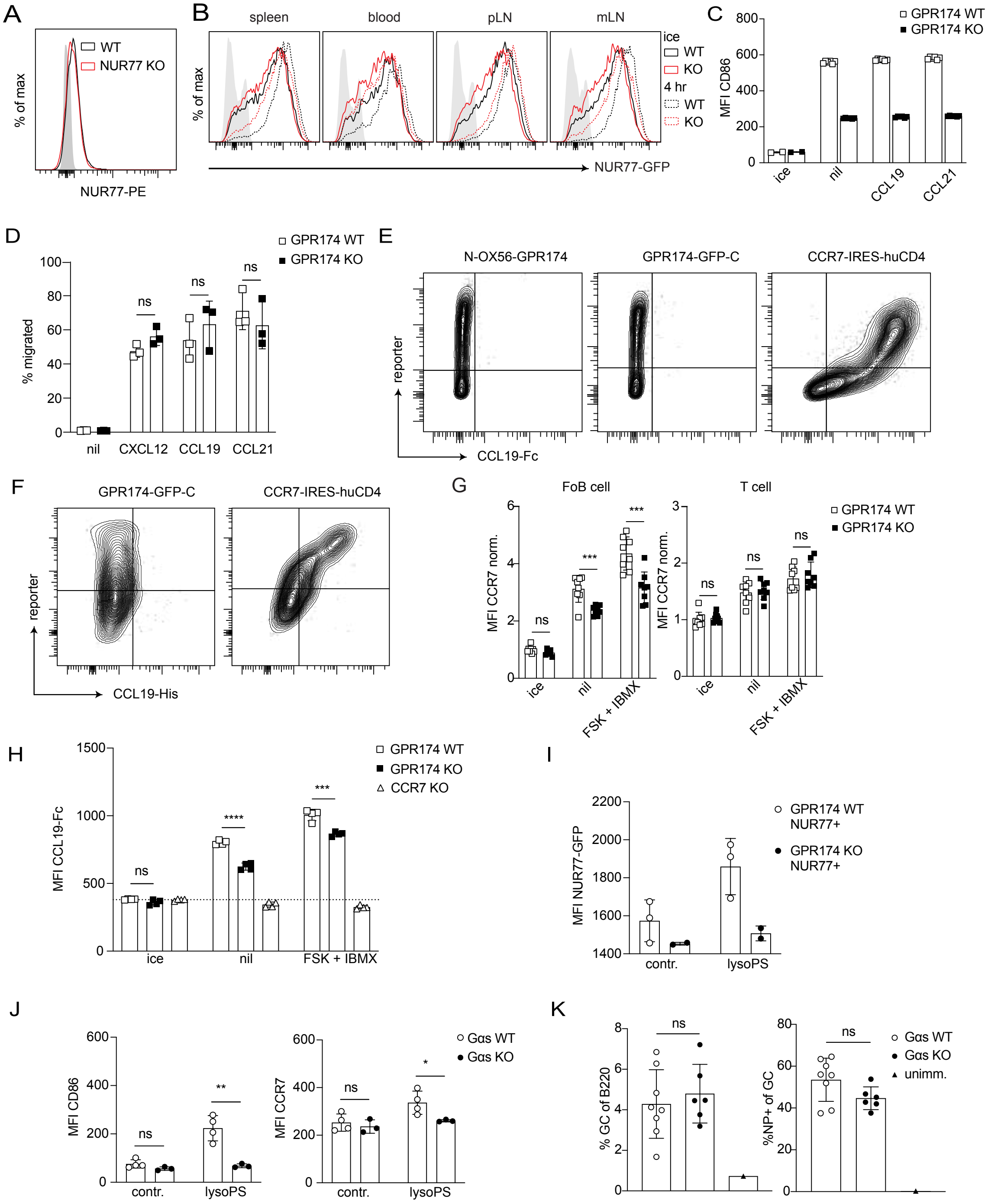

Fig. S5. GPR174 influences CCR7 and NUR77 expression in cultured B cells and following in vivo injection of lysoPS (A) Representative staining of intracellular NUR77 on WT versus NUR77 KO in freshly isolated B cells.(B) Histogram of NUR77-GFP on GPR174 WT (black) or KO (red) B cells from the indicated tissue either immediately after isolation (solid line) or after 4 hours of unstimulated culture (dashed line), compared to reporter negative (solid gray). (C) MFI of CD86 on B cells treated with or without CCL19 (500 ng ml^{-1}) or CCL21 (500 ng ml^{-1}) (triplicate wells of N=2 mice per genotype). (D) Percent of GPR174 WT and KO B cells migrated to CXCL12 (100 ng ml^{-1}), CCL19 (500 ng ml^{-1}), or CCL21 (500 ng ml^{-1}) during 3 hour assay following 48 hours of pre-activation with anti-IgM and anti-CD40 (10 μg ml^{-1}) (N=3 wells, representative of at least 3 individual experiments). (E) MFI of CCL19-Fc binding on HEK 293T cells transfected with N-terminally OX56 epitope tagged GPR174, C-terminally GFP tagged GPR174, or CCR7-ires-huCD4. Representative of 3 experiments. (F) MFI of CCL19-His binding on HEK 293T cells transfected with C-terminally GFP tagged GPR174, or CCR7-ires-huCD4. (G) MFI of CCR7 (normalized to WT B cells maintained on ice) on GPR174 WT and KO B cells (left) or T cells (right) after 6 hours of culture with or without forskolin + IBMX or maintained on ice (combined wells from two experiments of N=1 mice per genotype, representative of at least 3 additional experiments). (H) MFI of CCL19-Fc binding to follicular B cells from GPR174 WT, GPR174 KO, and CCR7 KO splenocytes cultured for 6 hours under the indicated conditions or maintained on ice. Dashed line indicates MFI of CCL19-Fc on CCR7 KO cells maintained on ice (N=4 wells per condition, representative of two experiments). (I) MFI of NUR77-GFP on GPR174 WT (N=3 mice) or KO (N=2 mice) B cells from popliteal lymph nodes after footpad injection of 25 μg 18:1 lysoPS or methanol (contralateral). (J) MFI of CD86 (left) or CCR7 (right) on $G\alpha$ WT (N=4 mice) or KO (N=3 mice) B cells from popliteal lymph nodes after footpad injection of 25 μg 18:1 lysoPS or methanol (contralateral). (K) Summary data of percent germinal center (GC, FAS^+GL7^+) of total B cells (left) and % NP^+ of GC (right) from spleens of $G\alpha s$ WT or KO mice immunized with 50 μg NP_{32}-KLH + 1 μg LPS in alum or an unimmunized control (unimm.). Data for (K) is representative of normal mice (N=2 WT and 4 KO) and chimeras (N=4 WT and 4 KO). Statistical significance for (D, G, H, J, K) determined by unpaired T-test. ns = not significant; *p < 0.05; **p < 0.01; ***p < 0.001; ****p < 0.0001.

SI Dataset S1 (DatasetS1.xlsx)

DEGs from pairwise comparisons of GPR174 WT and KO B cells cultured for 0 or 4 hours.

SI Dataset S2 (DatasetS2.xlsx)

DEGs between GPR174 WT and KO B cells cultured for 1 hour.

SI Dataset S3 (DatasetS3.xlsx)

DEGs from pairwise comparisons of Gαs WT and KO B cells cultured for 0 or 4 hours.
